# Salicylic Acid Treatment Ameliorates Postharvest Quality Deterioration in ‘France’ Prune (*Prunus domestica* L. ‘Ximei’) Fruit by Modulating the Antioxidant System

**DOI:** 10.3390/foods13182871

**Published:** 2024-09-10

**Authors:** Xinling Zhang, Yuxing Liu, Weida Zhang, Wanting Yang, Shuaibing An, Minrui Guo, Guogang Chen

**Affiliations:** 1 College of Food Science and Technology, Shihezi University, Shihezi 832000, China; 13239840759@163.com (X.Z.); liuyuxing233@163.com (Y.L.); zwd9411@163.com (W.Z.); 18139280260@163.com (W.Y.); 13461260105@163.com (S.A.); 2 Key Laboratory of Characteristics Agricultural Product Processing and Quality Control (Co-Construction by Ministry and Province), Ministry of Agriculture and Rural Affairs, School of Food Science and Technology, Shihezi University, Shihezi 832000, China; 3 Research Center of Xinjiang Characteristic Fruit and Vegetable Storage and Processing Engineering, Ministry of Education, Shihezi 832000, China

**Keywords:** ‘France’ prune, salicylic acid, fruit quality, oxygen species, antioxidant capacity

## Abstract

The potential of salicylic acid (SA) in delaying postharvest fruit senescence has been extensively documented; nevertheless, its effect on antioxidant activity and quality of ‘France’ prune fruit is largely unknown. The study investigated the effects of SA (0.5 mM) on postharvest quality deterioration of ‘France’ prune fruit. Results indicated that SA impeded the increase in respiration rate and weight loss, and mitigated the decrease of soluble solids content (SSC), titratable acidity (TA) content, firmness, and hue angle. SA sustained the ascorbate-glutathione cycle by inducing the production of ascorbic acid (AsA) and glutathione (GSH) and attenuates flavonoids, total phenols, and anthocyanins degradation by inhibiting polyphenol oxidase (PPO) activity and *PdPPO*. Moreover, SA significantly improved superoxide dismutase (SOD), catalase (CAT), ascorbate peroxidase (APX), peroxidase (POD), and glutathione reductase (GR) activities and gene expression levels, sustained higher 2,2′-Azinobis-(3-ethylbenzthiazoline-6-sulphonate) (ABTS) and 1,1-diphenyl-2-picryl-hydrazyl (DPPH) free radical scavenging capacity, ferric reducing antioxidant power (FRAP), and hydroxyl radical (·OH) inhibition capacity, and impeded the production of hydrogen peroxide (H_2_O_2_) and superoxide anion (O_2_^•−^). Overall, SA improved the antioxidant capacity by inducing the synthesis of defense response-related substances and promoting antioxidant enzyme activities to sustain the storage quality of ‘France’ prune fruit.

## 1. Introduction

‘France’ prunes (*Prunus domestica* L. ‘Ximei’) are a *Prunus* plant in the family Rosaceae that is native to West Asia and Europe and has been cultivated extensively in Xinjiang, China, in recent years. ‘France’ prune fruit has a rich aroma, a delicate and sweet taste, rich in various minerals and antioxidants, with cardiovascular system protection, bone health promotion, and constipation relief effects, among other nutritional benefits, making it extremely popular among consumers [1]. However, ‘France’ prunes are highly susceptible to dehydration, softening, and pathogenic microbial infestation following harvesting, which reduces the fruit’s nutritional quality, antioxidant activity, and storage life, with negative impacts on storage, transportation, and commercialization activities of ‘France’ prunes [2]. 

Salicylic acid (SA) is a low-cost, biodegradable, and efficient inducer for triggering the synthesis of biologically active metabolites, which are non-toxic to humans and environmentally friendly and have positive influences on the storage and nutritional characteristics of fruits and vegetables [3,4,5]. SA has been reported to act as endogenous signaling molecules involved in mediating physiological and biochemical processes, including antistress responses under biotic (microorganisms, pests, diseases, etc.) and abiotic (heavy metal, high or low temperature, and salinity stress) conditions, seed germination, growth, development, and fruit ripening [4]. Additionally, exogenous SA could induce the synthesis and accumulation of secondary metabolites (alkaloids, flavonoids, terpenoids, etc.) by regulating the antioxidant system and expression of various defense genes in plants, thus retarding ripening and aging of fruit and prolonging shelf life [6,7]. Gao et al. [8] found that SA could sustain cell membrane integrity and storage quality of post-harvest morels by improving antioxidant capacity. Zhang et al. [9] illustrated that SA mitigated the incidence of disease and senescence in fresh goji fruit by sustaining the content of hydrophilic and lipophilic bioactive components in the fruit. Gu et al. [10] demonstrated that SA mitigated the oxidative damage of reactive oxygen (ROS) on fruit cell membranes by inducing the accumulation of ascorbic acid (AsA), glutathione (GSH), and total phenols in sweet cherry fruit, thus effectively improving the fruit storage quality. Similar results were reported in postharvest SA-treated kiwifruit [11], citrus [12], and goji [13].

ROS is a class of harmful products inevitably produced during normal metabolic processes in plants and directly linked to postharvest senescence of fruit and vegetables [7]. During the storage of fruit and vegetables, the accumulation of ROS causes oxidative damage to proteins, lipids, carbohydrates, and DNA, directly inducing programmed cell death and accelerating the deterioration of quality [13]. Enzymatic and non-enzymatic antioxidant systems in fruits and vegetables play an indispensable role in mitigating cellular oxidative damage, maintaining redox homeostasis, and prolonging shelf life [8]. Previous studies demonstrated that exogenous SA attenuated postharvest leaf yellowing and senescence in Chinese flowering cabbage by enhancing antioxidant capacity and diminishing ROS production [14]. Apple polyphenols could ameliorate the postharvest browning of litchi fruit by promoting antioxidant enzyme activities and reducing the accumulation of malondialdehyde and ROS [15]. Therefore, postharvest regulation of ROS helps to improve storage quality and extend the shelf life of fruits and vegetables.

Considering the aforementioned beneficial effects of SA on postharvest physiological aspects of fruit and vegetables, the current study analyzed the alterations in physiological quality, bioactive component content, ROS accumulation, antioxidant-related enzyme activities, and related gene expression of ‘France’ prune fruit treated with SA during storage. This investigation will contribute to unraveling the regulatory mechanisms of SA on the antioxidant defense system in ‘France’ prune fruit during storage.

## 2. Material and Methods

### 2.1. Experimental Material Postharvest Treatment

‘France’ prune fruit were harvested from an orchard in Korla, Xinjiang, in September 2021, and sent immediately to the Fruit and Vegetable Storage and Preservation Research Center of Shihezi University. Fruit with similar maturity (Firmness: 2.5 ± 0.5 N; Soluble solids content [SSC]: 22.8 ± 0.5%), uniform size, free of pests and mechanical damage were selected and packed in foam boxes (100 fruit per box) and pre-cooled fully in a cold room at 4 ± 0.5 °C and a relative humidity of 85–90% for 24 h. After pre-cooling, the fruits were divided randomly into two groups: a control group (distilled water; CK) and a treatment group (0.5 mM SA, the SA (Source Leaf Biotechnology Co., Ltd., Shanghai, China) concentration was obtained through preliminary screening, as shown in Supplementary Figure S1).

Two groups of ‘France’ prune fruit were soaked in the corresponding solution for 5 min and air-dried naturally, then stored for 36 d at −1.5 ± 0.5 °C and a relative humidity of 85–90%. Samples were randomly taken every 6 days [1]. Among them, 90 fruit were selected randomly for each treatment (30 fruit as one biological replicate) for observation of weight loss, and 1050 fruit (150 fruit as 3 biological replicates for each sampling point of each treatment) for determination of the physiological quality parameters. After completion of these analyses, the remaining fruit was peeled and cut into pieces, frozen, and stored at −80 °C for use in later analyses.

### 2.2. Determination of Physiological Quality Parameters

The weight loss was calculated using the following formula: Weight loss (%) = ([A − B]/A) × 100, where A is the initial weight of the fruit (g) and B is the fruit weight (g) at the fruit sampling time. 

Fruit firmness (N) was measured using a firmness tester (GY-4, Yueqing Edelberg Instrument Co., Ltd., Yueqing, China) with a 3.5 mm probe. Randomly select 9 fruits and choose 3 locations on the equator of each fruit to measure the firmness after peeling. Repeat the measurement three times for each fruit.

The respiration rate (ng kg^−1^ s^−1^) of ‘France’ prune fruit was gauged using a fruit and vegetable respirometer (FS-3080A, Xinrui Instrument Co., Ltd., Shanghai, China). Fruit (40 fruit) was placed in a 2 L glass container and the data was recorded every 15 min for computation, and the experiment was repeated three times.

The a* (−green, +red) and b* (−blue, +yellow) values of the fruit were determined using a portable colorimeter (YS3060, Sanenshi Technology Co., Ltd., Shenzhen, China), and the hue angle was then computed using the formula: *h* = tan^−1^ (b*/a*). 

The SSC (%) of the ‘France’ prune fruit was measured using a portable refractometer (LB90T, Suwei Electronic Technology Co., Ltd., Guangzhou, China). 

The titratable acidity (TA) content of ‘France’ prune fruit was determined by acid-base titration, and the results were expressed as a percentage (%) of malic acid.

### 2.3. Determination of Bioactive Compounds

The AsA content was estimated using the method described by Gu et al. [10] with minor modifications. After homogenizing the pulp of a ‘France’ prune (10 g), it was made up to 100 mL with 5% trichloroacetic acid solution. After 10 min, the solution was filtered and 1.0 mL was taken in a test tube. Then, sequentially add 1.0 mL 0.5% trichloroacetic acid solution, 1.0 mL anhydrous ethanol solution, 0.5 mL 0.4% phosphoric acid-ethanol solution, and 1.0 mL 0.5% BP (4,7-diphenyl-1,10-phenanthroline)-ethanol solution, and finally add 0.5 mL 0.03% ferric chloride-ethanol solution to the test tube. The mixed solution was reacted at 30 °C for 60 min and the absorbance value was determined at 534 nm. Ascorbic acid content was expressed in g kg^−1^. 

Anthocyanin content (g kg^−1^) was calculated using the approach of Zhang et al. [15] with minor modifications. ‘France’ prune pulp (2 g) was mixed and homogenized with 10 mL 80% methanol solution and extracted by ultrasonication (40 kHz) for 1 h. Afterward, 1 mL of the extract was combined with a potassium chloride buffer and a sodium acetate buffer, respectively, then the absorbance values of the solutions were determined at 510 nm and 700 nm after being left to stand for 15 min. The anthocyanin content was computed using the formula, where A is the absorbance value, M_w_ is the relative molecular mass of Cyanidin-3-galactoside, DF is the dilution ratio, ε is the molar absorptivity of Cyanidin-3-galactoside, and *L* is the optical path length of the cuvette.

Anthocyanin content (g∙kg^−1^) = (A × M_w_ × DF)/(ε × *L*) (1)

A = (A_510_ − A_700_)pH_1.0_ − (A_510_ − A_700_)pH_4.5_, M_w_ = 449.2 g mol^−1^, DF = 1, ε = 26,900 L (mol·cm)^−1^, *L* = 1 cm.

Flavonoids and total phenolics contents (g kg^−1^) were calculated using the approach of Yang et al. [7]. Pulp samples (5 g) were mixed with 15 mL 80% methanol solution, homogenized, and then extracted by sonication for 30 min. The supernatant was mixed and reduced to 50 mL after three replicates. The absorbance values of the extracts were calculated at 760 nm and 510 nm and substituted in the standard curves of gallic acid and rutin, respectively, to calculate the contents of total phenolics and flavonoids, respectively.

The GSH content in ‘France’ prune fruit was measured with a kit (Suzhou Grace Biotechnology Co., Ltd., Suzhou, China). The frozen samples were weighed at 0.1 g and homogenized in an ice bath by adding 0.1 mL of extraction solution, followed by the sequential addition of the corresponding reagents according to the instructions in the kit, and the absorbance value of the reaction solution was measured at 412 nm. The results were expressed as μmol·kg^−1^.

### 2.4. Determination of ROS Levels

The hydroxyl radical (·OH) inhibition capacity in ‘France’ prune pulp was calculated using a kit (Nanjing Jiancheng Institute of Biological Engineering, Nanjing, China). Added 0.9 mL of saline to 0.1 g of sample, homogenized and centrifuged, and then added the reagents provided in the kit sequentially according to the instructions in the kit, and measured the absorbance value of the reaction solution at 550 nm. The results were expressed as U kg^−1^. 

The hydrogen peroxide (H_2_O_2_) and superoxide anion (O_2_^•−^) content were determined with reference to the experimental instructions provided by the kit (Suzhou Keming Biotechnology Co., Ltd., Suzhou, China). Take 0.1 g of the sample separately in an EP tube and extract the samples according to the instructions provided in the H_2_O_2_ and O_2_^•−^ content kits. The absorbance values of the reaction solutions were measured at 415 nm and 530 nm, respectively. The results were expressed as μmol kg^−1^.

### 2.5. Determination of Antioxidant Enzyme Activity

Superoxide dismutase (SOD) and catalase (CAT) activities were measured using SOD and CAT detection kits (Suzhou Keming Biotechnology Co., Ltd., Suzhou, China). The sample was weighed at 0.1 g, respectively, and the supernatant was prepared according to the instructions provided in the SOD and CAT kits. The SOD and CAT activities in the samples were determined according to the instructions, respectively. The results were expressed as U kg^−1^.

Ascorbate peroxidase (APX) and glutathione reductase (GR) activities were assessed following the approach of Zhang et al. [16]. APX enzyme activity was defined based on the catalytic oxidation of 1 μmol of AsA per second per kilogram of sample, whereas GR enzyme activity was defined based on the catalytic oxidation of 1 μmol NADPH per second per kilogram of sample. 

Peroxidase (POD) activity was calculated using the approach suggested by Xu et al. [17], where each kg of tissue increased the absorbance at 470 nm by 0.5 per min in the reaction system as one unit of enzyme activity. 

Polyphenol oxidase (PPO) activity was analyzed following the instructions of the PPO kit (Suzhou Grace Biotechnology Co., Ltd., Suzhou, China). PPO activity was defined as the change in absorbance values of 0.005 at 420 nm per minute per kilogram of tissue in the reaction system. The enzyme activity units were expressed with U kg^−1^.

### 2.6. Analysis of Antioxidant Enzyme Gene Expression

The total RNA of ‘France’ prune pulp was extracted using an all-purpose plant RNA extraction kit (Kangwei Century Biotechnology Co., Ltd., Taizhou, China). The total RNA concentration in the extracts was detected using an ultraviolet spectrophotometer (NANO 2000, Thermo Fisher Scientific, Waltham, MA, USA). Template RNA (1 μg) was obtained for reverse transcription and then specific primers encoding ‘France’ prune SOD, PPO, GR, CAT, POD, and APX genes were designed using Primer Premier 5.0 software (PREMIER Biosoft, San Francisco, CA, USA) (Table 1). The quantitative real-time PCR (qRT-PCR) analysis was conducted using the ExicyclerTM 96 PCR instrument (BIONEER Biotechnology Co., Ltd., Daejeon, Republic of Korea). The PCR reaction conditions were 95 °C for 5 min, followed by 40 cycles at 95 °C for 10 s, 60 °C for 10 s, and 72 °C for 15 s. Following a melting temperature cycle, fluorescence data were collected continuously in the range of 60–94 °C for subsequent analysis. The quantification level of each gene was calculated using the 2^−ΔΔCT^ method. Actin was used as an internal reference gene owing to its high expression and stability. All samples were subjected to three sets of parallel experiments per gene.

### 2.7. Determination of Antioxidant Capacity

The DPPH free radical scavenging capacity (%) was determined based on the method described by Kenny and O’Beirne [18]. After ultrasonic extraction of 5 g pulp and 15 mL 80% methanol solution homogenate for 30 min. The supernatant (0.25 mL) was mixed evenly with 1.5 mL methanol and 2 mL DPPH solution and left to stand in the darkness for 30 min. Afterward, its absorbance value was determined at 517 nm.

The ABTS free radical scavenging capacity (%) was ascertained using the approach of Jeong et al. [19]. The ABTS (7 mmol L^−1^) solution was combined with 2.45 mmol L^−1^ potassium peroxydisulfate solution in equal volumes and reacted in the dark for 12 h. The solution was diluted with 70% ethanol solution until the absorbance value was maintained within 0.70 ± 0.02 range. The sample solution (0.2 mL) and the reserve solution (3.8 mL) were mixed uniformly and reacted at ambient temperature for 10 min. The absorbance value of the reaction solution was recorded at 734 nm.

The total antioxidant capacity was determined using the ferric-reducing ability of a plasma (FRAP) assay kit (Beijing Box Biotechnology Co., Ltd., Beijing, China). The results were expressed in mmol kg^−1^.

### 2.8. Statistical Analysis

All the aforementioned experiments were repeated three times in parallel and then averaged. Plotting was performed using OriginPro (version 2020b, Origin Lab Corp., Northampton, MA, USA). A one-way analysis of variance (ANOVA) was conducted among groups within each time point in IBM SPSS Statistics 24 (IBM Corp., Armonk, NY, USA). *p* < 0.05 indicated that the differences between treatment groups were significant. Pearson’s correlation test was employed to assess the correlations among indicators, and the correlation heat map was drawn using ChiPlot (https://www.chiplot.online/, accessed on 29 May 2024).

## 3. Results

### 3.1. Physiological Quality Parameters

**Figure 1:**
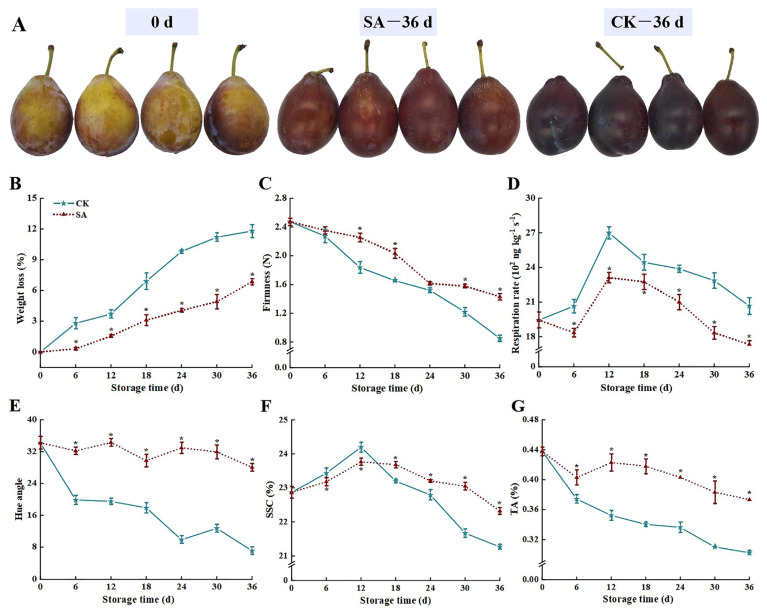
Effects of salicylic acid (SA) treatment on postharvest sensory changes (**A**), weight loss (**B**), firmness (**C**), respiration rate (**D**), hue angle (**E**), SSC (**F**), and TA (**G**) of ‘France’ prune fruit. Error bars represent the standard deviation of the mean after three replicates for each set of data. The asterisk indicated a significant difference between the treatment group and the CK group for the same storage days (*p* < 0.05). SSC, soluble solid content; TA, titratable acidity.

As illustrated in Figure 1A, the peel of ‘France’ prune fruit had an obvious green color at 0 d, and at 36 d of storage, the peel of CK the group showed a dark red color and the fruit stalks appeared to obviously drop, while the peel of the SA group showed bright red color. By measuring physiological parameters, we found that SA effectively sustained the firmness, hue angle, SSC, and TA of ‘France’ prune fruit while demonstrating lower weight loss and a lower respiration rate compared to the CK group (Figure 1B–G). At 36 d, the firmness, hue angle, SSC, and TA of the SA group were 68.2%, 294.9%, 5.0%, and 23.4% higher than those of the CK group, respectively, whereas weight loss and respiration rate were 42.0% and 16.1% lower than those of the CK group, respectively (*p* < 0.05).

### 3.2. Bioactive Components

**Figure 2:**
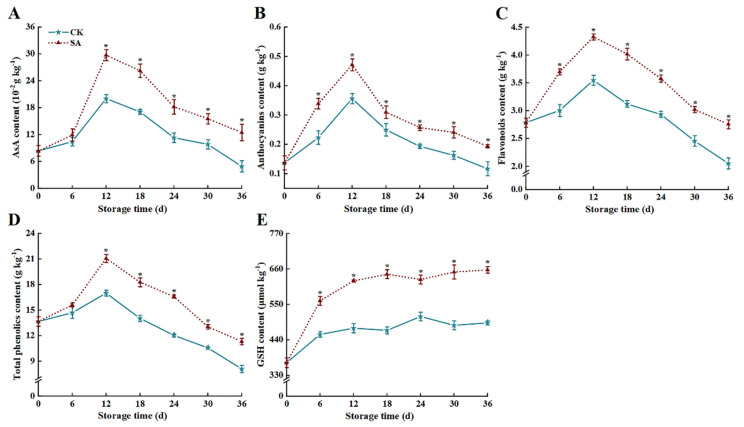
Effects of salicylic acid (SA) treatment on postharvest AsA (**A**), anthocyanins (**B**), flavonoids (**C**), total phenolics (**D**), and GSH (**E**) of ‘France’ prune fruit. Error bars represent the standard deviation of the mean after three replicates for each set of data. The asterisk indicated a significant difference between the treatment group and the CK group for the same storage days (*p* < 0.05). AsA; ascorbic acid, GSH; glutathione.

The trends of AsA, anthocyanins, flavonoids, and total phenolic contents in the two groups during storage were similar, peaking at 12 d and then decreasing. SA treatment effectively impeded the decrease of AsA, anthocyanins, flavonoids, and total phenolic contents, and was 153.9%, 65.8%, 34.5%, and 39.8% higher than that of the CK group at 36 d, respectively (*p* < 0.05) (Figure 2A–D). Meanwhile, SA promoted the accumulation of GSH contents, which was 33.5% higher than that of the CK group (*p* < 0.05) (Figure 2E).

### 3.3. ROS Levels

**Figure 3:**
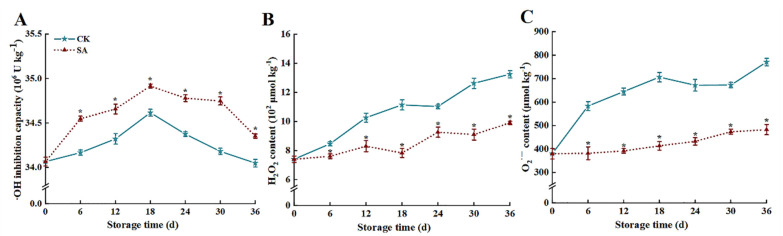
Effects of salicylic acid (SA) treatment on postharvest ·OH inhibition capacity (**A**), H_2_O_2_ (**B**), and O_2_^•−^ (**C**) of ‘France’ prune fruit. Error bars represent the standard deviation of the mean after three replicates for each set of data. The asterisk indicated a significant difference between the treatment group and the CK group for the same storage days (*p* < 0.05). ·OH, hydroxyl radical; H_2_O_2_, hydrogen peroxide; O_2_^•−^, superoxide anion.

As illustrated in Figure 3A, the ·OH inhibition capacity of ‘France’ prune fruit decreased continuously after 18 d. However, SA treatment impeded the decline of ·OH inhibition capacity. At 36 d, the contents of H_2_O_2_ and O_2_^•−^ were 25.3% and 37.3% lower in the SA group than those of the CK group, respectively (*p* < 0.05) (Figure 3B,C).

### 3.4. Antioxidant Enzyme Activity

**Figure 4:**
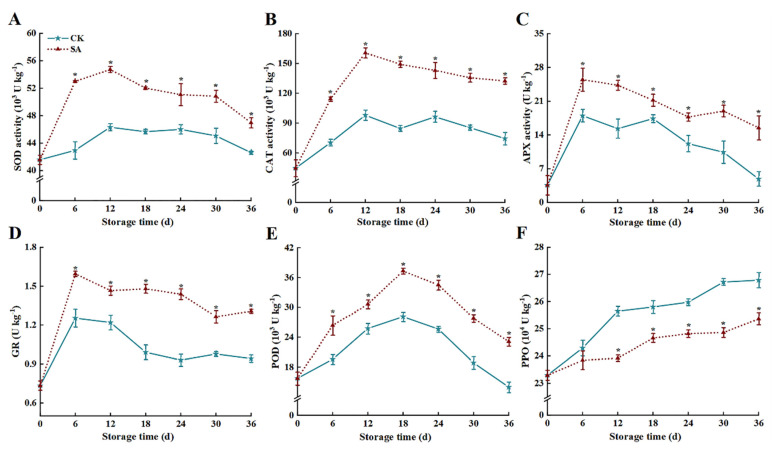
Effects of salicylic acid (SA) treatment on postharvest SOD (**A**), CAT (**B**), APX (**C**), GR (**D**), POD (**E**), and PPO (**F**) activities of ‘France’ prune fruit. Error bars represent the standard deviation of the mean after three replicates for each set of data. The asterisk indicated a significant difference between the treatment group and the CK group for the same storage days (*p* < 0.05). SOD, superoxide dismutase; CAT, catalase; APX, ascorbate peroxidase; GR, glutathione reductase; POD, peroxidase; PPO, polyphenol oxidase.

The SOD and CAT activities of both groups increased continuously during the pre-storage period (0–12 d), followed by a gradually diminishing trend (Figure 4A,B). At 36 d, SOD and CAT activities in the SA group were 10.2% and 53.7% higher than the CK group, respectively (*p* < 0.05). APX and GR activities increased rapidly during the first 6 d, followed by a continuous reduction from 6 to 36 d (Figure 4C,D). However, SA sustained high APX and GR activities, which were 216.9% and 38.7% greater than those of the CK group, respectively (*p* < 0.05). POD activities in the two groups peaked at 18 d, and the POD activity of the SA group was 1.33 times higher than that of the CK group. At 36 d, the POD activity of the SA group was 65.2% higher than that of CK (*p* < 0.05) (Figure 4E). 

Unlike the former, SA has an inhibitory effect on the enhancement of PPO activity (Figure 4F). At 36 d, the PPO activity of the SA group was 5.3% lower than that of the CK group (*p* < 0.05).

### 3.5. Antioxidant Enzyme Gene Expression

**Figure 5:**
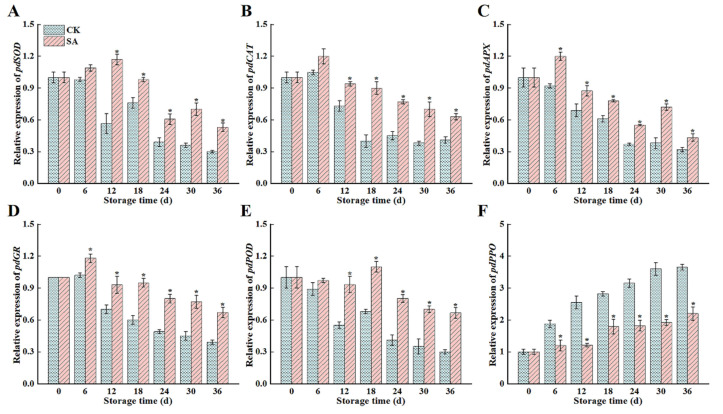
Effects of salicylic acid (SA) treatment on postharvest *PdSOD* (**A**), *PdCAT* (**B**), *PdAPX* (**C**), *PdGR* (**D**), *PdPOD* (**E**), and *PdPPO* (**F**) gene expression levels of ‘France’ prune fruit. Error bars represent the standard deviation of the mean after three replicates for each set of data. The asterisk indicated a significant difference between the treatment group and the CK group for the same storage days (*p* < 0.05).

To elucidate antioxidant enzyme activity trends in ‘France’ prune fruit after SA treatment, the expression levels of key genes, *PdSOD*, *PdCAT*, *PdAPX*, *PdGR*, *PdPOD*, and *PdPPO*, were analyzed using qRT-PCR. As demonstrated in Figure 5, the expression levels of *PdSOD*, *PdCAT*, *PdAPX*, *PdGR*, and *PdPOD* in the two groups were observed to gradually improve and then reduce during the storage period, and the expression of the genes in the CK group was always lower than that in the SA group (Figure 5A–E). SA impeded the enhancement of *PdPPO* gene expression, and compared with the CK group, the expression level of the *PdPPO* gene decreased by 39.8% (*p* < 0.05) (Figure 5F).

### 3.6. Antioxidant Capacity

**Figure 6:**
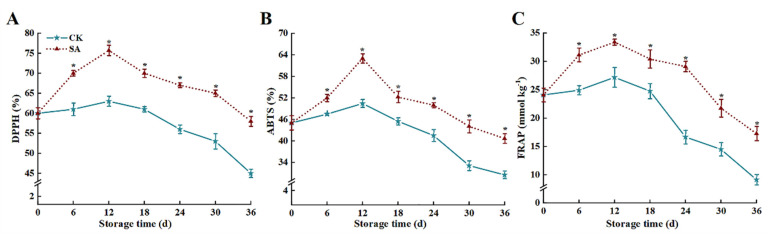
Effects of salicylic acid (SA) treatment on postharvest DPPH (**A**), ABTS (**B**), and FRAP (**C**) of ‘France’ prune fruit. Error bars represent the standard deviation of the mean after three replicates for each set of data. The asterisk indicated a significant difference between the treatment group and the CK group for the same storage days (*p* < 0.05). DPPH, DPPH free radical scavenging capacity; ABTS, ABTS free radical scavenging capacity; FRAP, ferric reducing antioxidant power.

SA sustained higher levels of antioxidant capacity in ‘France’ prune fruit compared to the CK group (Figure 6). At 36 d of storage, the DPPH (Figure 6A), ABTS (Figure 6B), and FRAP (Figure 6C) of the SA group fruit were 28.9%, 33.2%, and 88.8% higher than those of the CK group, respectively (*p* < 0.05).

### 3.7. Correlation Analysis

**Figure 7:**
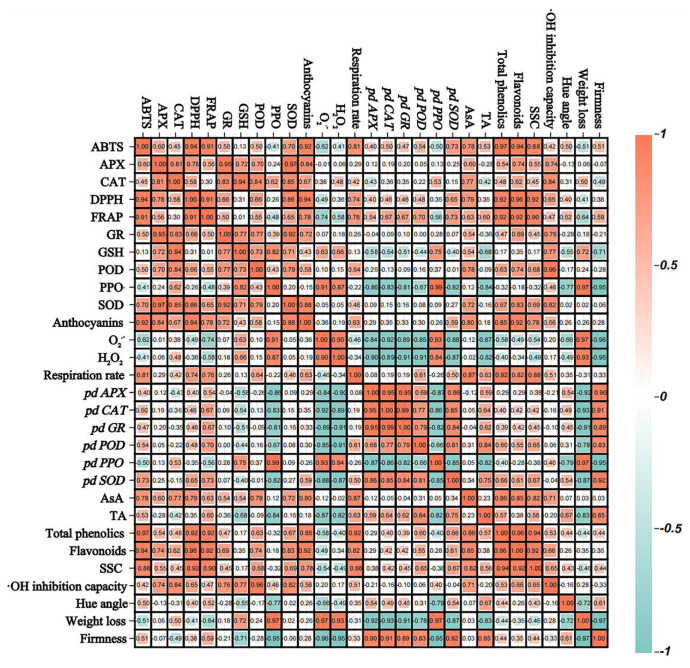
Correlation of indicators in the antioxidant defense system of ‘France’ prune fruit in the SA group. Blue indicates a negative correlation and red indicates a positive correlation.

As shown in Figure 7, fruit firmness, TA, SSC, and hue angle were negatively correlated with H_2_O_2_ and O_2_^•−^, and weight loss was positively correlated with H_2_O_2_ and O_2_^•−^. In contrast, antioxidant capacity (ABTS, DPPH, and FRAP), total phenolics, and flavonoids were positively correlated with firmness and negatively correlated with H_2_O_2_ and O_2_^•−^. That is, antioxidant system-related indices affect ROS production, which potentially influences the overall storage quality of ‘France’ prune fruit. Notably, ABTS, DPPH, and FRAP were positively correlated with CAT, APX, GR, POD, SOD, anthocyanins, AsA, total phenolics, and flavonoids.

## 4. Discission

‘France’ prune, as a typical respiratory climacteric fruit, will soften, rot, and wrinkle even when stored in a low-temperature environment, which reduces the quality and nutritional benefits of the fruit considerably [1]. SA, as a signaling molecule, can regulate plant growth and development, as well as various physiological and metabolic activities by regulating gene expression and increasing antioxidant levels in postharvest fruit, thereby sustaining the storage quality of fruits and vegetables [20]. Therefore, the current study used exogenous SA (0.5 mM) to treat ‘France’ prune fruit and explore its effects on storage quality and antioxidant capacity to determine safe and effective storage and preservation methods for extending the storage life of ‘France’ prune fruit.

Weight loss and firmness are representative indicators of fruit freshness and consumer acceptability [1], and respiration intensity can reflect the life stage of fruit in addition to storage tolerance and quality changes in fruit and vegetables [21]. In the current study, 0.5 mM SA treatment impeded an increase in the respiration rate of ‘France’ prune fruit and sustained higher firmness as well as lower weight loss (Figure 1). Rasouli et al.’s study is similar to our findings, which suggest that SA can maintain the firmness of citrus fruit by affecting cell turgor pressure and morphology [22]. Serna-Escolano et al. [23] indicated that suppression of fruit respiratory metabolism was effective in retarding the increase in substrate consumption and weight loss rate, hence sustaining the cellular morphology and firmness of the fruit. Therefore, we concluded that SA could mitigate postharvest softening and weight loss by impeding the respiratory rate of ‘France’ prune fruit. The color of the fruit peel reflects the maturity of the fruit and is an essential indicator for judging the degree of fruit senescence [24]. The hue angle of the ‘France’ prune fruit exhibited an overall downward trend during the storage period, indicating that the color difference of the peel increased gradually, and the fruit color changes from initially bright to dark, marking the transition of the fruit from maturity to aging. Compared with the CK group, SA mitigated the decline of fruit hue angle, indicating that SA treatment facilitated maintenance of the color of ‘France’ prune fruit.

SSC and TA are the key factors influencing fruit flavor and quality, and changes in SSC and TA content caused by fruit respiration and glucose metabolism could reflect the metabolic states, degree of maturity, and senescence of fruit [25,26]. This study revealed that SSC in the CK and SA groups continued to accumulate during the first 12 days of storage, attributed to the gradual degradation of macromolecular substances such as starch polysaccharides into soluble monosaccharides and small molecular substances during the early stages of storage [27]. After 12 days of storage, SSC began to decrease due to the consumption of a large amount of soluble sugars and carbohydrates to satisfy the needs of respiratory metabolism and fruit repair mechanisms [28]. SA sustained higher SSC in ‘France’ prune fruit, which corresponded to the lower respiration rate, indicating that treatment could impede fruit respiratory metabolism and reduce substrate consumption, thereby sustaining fruit postharvest quality. TA is a key factor influencing fruit flavor [29]. The decreasing trend of TA in both groups of fruit may be attributed to (1) the synthesis of organic acids being impeded by fruit ripening and senescence; (2) organic acids as respiratory substrates are consumed constantly, oxidized and decomposed into carbon dioxide, water, and energy during glycolysis and the tricarboxylic acid cycle [30]; (3) organic acids are involved in metabolic processes, such as the synthesis of phenols, amino acids, esters, and aromatic substances [7]; (4) some organic acids combine with metal ions, such as potassium and calcium, to form organic acid salts, which reduces organic acid contents in cells [9]. Remarkably, the SA group had higher TA levels than the CK group (Figure 1F) due to SA reducing the respiration intensity of fruit, thus diminishing the consumption of organic acids as substrates for respiratory metabolism. Similar results were observed in SA-treated blueberries [31], winter jujubes [7], goji berries [9], and oranges [30].

ROS is an essential signaling substance for numerous major physiological processes in plants as well as a toxic by-product of aerobic metabolism. ROS was used as an indicator for measuring the degree of oxidative damage in fruits and vegetables [32]. When fruit and vegetables are exposed to biotic or abiotic stress, ROS in the forms of ·OH, H_2_O_2_, O_2_^•−^, etc., are continuously produced as oxidative bursts in vivo, and the low amounts of ROS produced in the early stages of the oxygen bursts can serve as signaling molecules to activate related defense responses [32]. When equilibrium is disrupted, excessive ROS production will trigger oxidative stress, leading to postharvest respiratory metabolism disorders and irreversible oxidative damage to the cell structure, resulting in fruit browning, senescence, and softening [1,33]. Enzymatic (SOD, CAT, POD, APX, GR, etc.) and non-enzymatic (GSH, AsA, etc.) antioxidant defense systems in fruit play an indispensable role in retarding senescence, browning, and oxidative damage [34]. SOD disproportionately dismutates O_2_^•−^ to generate H_2_O_2_, while CAT synergizes with POD in degrading H_2_O_2_ to non-toxic H_2_O and O_2_ [1]. GSH and AsA could be directly involved in ROS scavenging as reductants, and constitute crucial AsA-GSH cycling systems alongside GR and APX enzymes to sustain intracellular redox homeostasis and diminish oxidative stress [7,35,36]. Siboza et al. [20] found that SA impeded ROS accumulation by stimulating the activities of CAT, APX, and GR, and provided more reducing power for lemon fruit, effectively mitigating oxidative damage of fruit cell membranes and chilling injury incidence. Gu et al. [10] found that SA could impede ROS accumulation in sweet cherry fruit by increasing antioxidant enzyme activities, thereby sustaining fruit storage quality. Zhang et al. [13] indicated that SA can mitigate the oxidative damage of cell membranes and ROS accumulation by increasing antioxidant enzyme activity (CAT, SOD, APX, etc.) and related gene expression in goji berries, thereby maintaining the quality of goji berries. In the present study, SA treatment sustained higher levels of activities and relative gene expression for SOD, CAT, POD, APX, and GR, mitigated the reduction of AsA and GSH contents, improved ·OH inhibition capacity and impeded ROS (H_2_O_2_ and O_2_^•−^) accumulation. This will help maintain the antioxidant balance and quality of ‘France’ prune fruit.

Anthocyanin, total phenolics, flavonoids, and other bioactive components act as electron donors to participate in and scavenge surplus ROS generated in fruit, and facilitate antioxidant homeostasis in fruit [9]. Previous research indicates that SA could rapidly and selectively induce the expression of specific genes with specific biochemical signals, thereby regulating the synthesis of secondary metabolites (phenolics, flavonoids, terpenoids, etc.) in plant cells to mitigate oxidative stress and damage caused by chilling injury [13,37]. In this study, we found that the total phenolic, flavonoid, and anthocyanin contents of SA-treated fruit increased rapidly and were higher than those of the CK group during the whole storage period (Figure 2). The aforementioned statement is consistent with the findings of Jiang et al. [31], who verified that SA as exogenous signaling molecules could induce the accumulation of bioactive components in postharvest blueberry fruit. In addition, Zhang et al. [9] also found that SA treatment could increase the total phenolics, flavonoids, AsA, and carotenoids contents of fresh goji fruit and maintain the antioxidant capacity and storage quality of the fruit. However, from 12 to 36 d, the anthocyanin, total phenolics, and flavonoid contents of the SA and CK groups exhibited diminishing trends, probably attributed to their unstable and easily degradable properties [30,38]. Studies have found that with the aggravation of fruit senescence, damage to the cell membrane system will cause cellular compartmentalized structures to be destroyed, prompting PPO to catalyze phenolic oxidation into quinones and further oxidation and polymerization to form brown products, which induce browning and deterioration of fruit tissue quality [15]. In the present study, SA sustained low levels of PPO activity and *PdPPO* expression in ‘France’ prune fruit at the end of storage, which explained the reason for the higher phenolic compounds in this group. Sinha et al. [39] found that SA combined with chitosan effectively impeded postharvest pear fruit PPO activity and diminished the degradation of phenolic compounds and browning degree in fruit, which was consistent with our conclusion. 

The ABTS, DPPH, and FRAP assays are widely utilized for the assessment of the antioxidant capacity of fruits and vegetables. Studies have indicated that the antioxidant capacity of plants depends primarily on the type and content of bioactive components and antioxidant enzyme activities [40]. In this research, the SA group had higher ABTS, DPPH, and FRAP compared with the CK group (Figure 6), which is related to the ability of SA to maintain high levels of total phenols, flavonoids, and anthocyanins; improve fruit antioxidant enzyme activity and gene expression; and maintain the balance of the fruit antioxidant system. Correlation analysis also observed a positive association between ABTS, DPPH, and FRAP levels in the SA group with total phenolics, flavonoids, and anthocyanins, respectively (Figure 7). Fan et al. [41] revealed that the sustainment of total phenolics and flavonoid contents contributed to enhancing the antioxidant capacity of apricot fruit and were positively correlated with ABTS, DPPH, and FRAP. Furthermore, Zhang et al. [9] also confirmed a positive correlation between the levels of bioactive compounds (total phenols, flavonoids, anthocyanins) and the antioxidant capacity in postharvest goji fruit. In conclusion, SA treatment could enhance the antioxidant capacity of ‘France’ prune fruit by inducing the activity of antioxidant enzymes and modulating the expression of related genes, while stimulating bioactive component accumulation, thereby curbing excessive ROS accumulation and postharvest quality deterioration in ‘France’ prune fruit.

## 5. Conclusions

**Figure 8:**
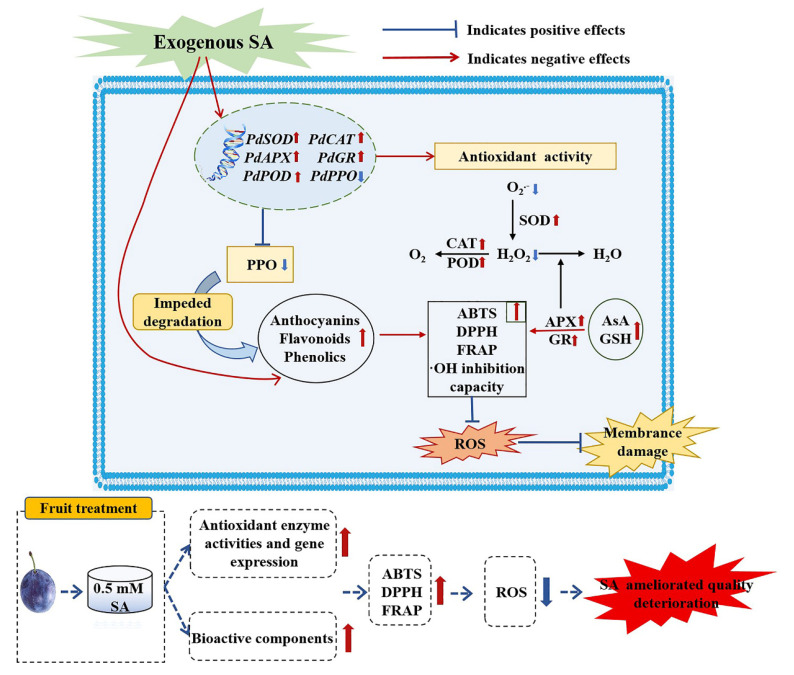
Mechanism of SA treatment in retarding quality deterioration of ‘France’ prune fruit by enhancing antioxidant defense system.

Based on the findings of our study, SA enhanced SOD, CAT, APX, POD, and GR activities and related gene expression, as well as the accumulation of bioactive components (AsA, GSH, flavonoids, total phenolics, and anthocyanins). In addition, SA impeded the increase in PPO activity and *pdPPO* expression; increased ABTS, DPPH, FRAP, and ·OH inhibition capacity of ‘France’ prune fruit during storage; and mitigated ROS (H_2_O_2_ and O_2_^•−^) production. And SA could retard the increase in weight loss, sustain firmness, hue angle, SSC, and TA content of ‘France’ prune fruit by diminishing respiration rate (Figure 8). Therefore, SA could be applied as an effective and feasible postharvest storage and preservation technology to maintain the postharvest storage quality of ‘France’ prunes.

## Figures and Tables

**Table 1:** Primer sequences for qRT-PCR.

Gene	Gene ID	Forward Primer (5′–3′)	Reverse Primer (5′–3′)
Actin	103339527	TCAACCCTAAGGCAAAC	GTGGCTGACACCATCTC
*PdSOD*	103339036	TTCAGATAAGGAGCGTCAC	CATTCCCAGTAGTCTTGCT
*PdPPO*	103324677	CCGCACAGGCATAAGCAC	GGCACCAAAGTCACCACC
*PdGR*	103324330	GGCTGTCGGTGATGTTA	TTTGGCTTGTTCTATTGC
*PdCAT*	103336676	CAGGGAAAGCACAGTAT	TCAAGTGGGTCAAAGTC
*PdPOD*	103342243	TCCTCCCACTTCTACCC	ACATTTGCTTTGCCTGA
*PdAPX*	103340411	GGGTTGTTGCTGTGGAG	CCTTGTTTGGCATCTGG

## Data Availability

The original contributions presented in the study are included in the article/Supplementary Material, further inquiries can be directed to the corresponding author(s).

## Supplementary Materials

The following supporting information can be downloaded at: https://www.mdpi.com/article/10.3390/foods13182871/s1, Figure S1: Effects of different concentrations of SA treatment on postharvest weight loss (A) and firmness (B) of ‘France’ prune fruit. Different superscript letters (a–d) indicate significant differences between the same storage days (*p* < 0.05).
