# Supplementary material for: Salicylic Acid Treatment Ameliorates Postharvest Quality Deterioration in ‘France’ Prune (Prunus domestica L. ‘Ximei’) Fruit by Modulating the Antioxidant System
Source: Foods. 2024 Sep 10;13(18):2871. doi: 10.3390/foods13182871 (PMC11430936; doi:10.3390/foods13182871)
Supplement: Supplementary file 1 [file foods-13-02871-s001.zip › figure S1.pdf]

Supplementary Material

Salicylic Acid Treatment Ameliorates Postharvest Quality Deterioration in 'France' Prune (*Prunus domestica* L. 'Ximei') Fruit by Modulating the Antioxidant System

Xinling Zhang [1,2], Yuxing Liu [1,2], Weida Zhang [1,2], Wanting Yang [1,2], Shuaibing An [1,2], Minrui Guo [1,2,3,*] and Guogang Chen [1,2,3,*]

1 College of Food Science and Technology, Shihezi University, Shihezi 832000, China; 13239840759@163.com (X.Z.); liuyuxing233@163.com (Y.L.); zwd9411@163.com (W.Z.); 18139280260@163.com (W.Y.); 13461260105@163.com (S.A.)

2 Key Laboratory of Characteristics Agricultural Product Processing and Quality Control (Co-Construction by Ministry and Province), Ministry of Agriculture and Rural Affairs, School of Food Science and Technology, Shihezi University, Shihezi 832000, China

3 Research Center of Xinjiang Characteristic Fruit and Vegetable Storage and Processing Engineering,
Ministry of Education, Shihezi 832000, China

* Correspondence: gmrshzu@163.com (M.G.); cgg611@163.com (G.C.)

Supplementary Figure S1

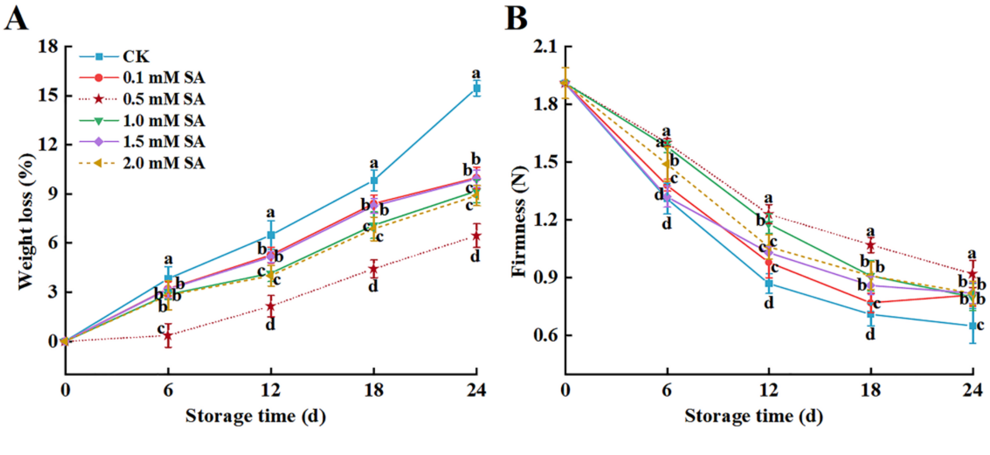

Supplementary Figure S1. Effects of different concentrations of SA treatment on postharvest weight loss (A) and firmness (B) of 'France' prune fruit. Different superscript letters (a-d) indicate significant differences between the same storage days (p<0.05).
